# A Case of the Cure of Cancer of Both Breasts, the One Ulcerated, the Other Schirrous

**Published:** 1800-10

**Authors:** William Nisbet

**Affiliations:** Fellow of the Royal College of Surgeons, Edinburgh; now of London: and Mr. Isaac Oliphant, Member of the Royal College of Surgeons, London


### *A Case of the Cure of Cancer of both Breasts, the one ulcerated, the other schirrous.*

By William Nisbet, M.D. Fellow of the Royal College of Surgeons, Edinburgh; now of London: and Mr. Isaac Oliphant, Member of the Royal College of Surgeons, London.

### *To the Editors of the Medical and Phyſical Journal.*

Gentlemen,

Having devoted my attention of late years to a particular line of medical practice, the ſubject of which has been deemed one of the chief opprobria of the profeſſion; and from a conviction that this general opinion is by no means well founded, and that Cancer is equally curable as any other ſpecies of ſwelling or ulcer, it is incumbent on me, in entertaining ſuch new ſentiments, to ſupport them by the evidence of incontrovertible facts.—I ſhall therefore ſtate the following caſe, one of the moſt melancholy that can occur, as an introduction to a number of others which ſhall be occaſionally given to the public, through the medium of your uſeful Miſcellany.

Mrs. G. the wife of Mr. G. a coal merchant in Tottenham Court Road, aged upwards of 40, about two years ago, felt a hardneſs and ſwelling of her left breaſt, with all the uſual ſymptoms of ſchirrous or incipient cancer. For this complaint ſhe firſt conſulted Mr. Ford, who adviſed, in that ſtage of the diſeaſe, the application of leeches, which was done without effect. She then put herſelf under the care of Mr. Cline, and continued to follow his preſcriptions for the ſpace of two months, not only with equal inefficacy, but with a gradual aggravation and increaſe of the diſeaſe, which was attended with the moſt ſevere darting pains and burning heat. In this unhappy ſtate, anxious to obtain relief, her next reſort was to the advice of Sir James Earle, who told her that it was a ſtony cancer, and incurable, declining at the ſame time to preſcribe any thing, but humanely offering (being ignorant of her real circumſtances in life) to give her a letter of admiſſion to the Middleſex Hoſpital. At the requeſt of Dr. Budd, to whom her huſband had been recommended, ſhe was next ſeen by Mr. Cooper, who concurred in opinion with Sir J. Earle, that it was incurable, and declined any connexion with the caſe. Thus deſerted by the Faculty, the patient was induced, by the over-perſuaſion of friends, to put herſelf into the hands of an ignorant empyric, under whoſe applications the diſeaſe paſſed rapidly into the ſtage of ulceration, and made horrid progreſs. In conſequence of the ſeverity of her ſufferings, ſhe was ſoon again obliged to ſolicit the aſſiſtance of a regular practitioner, and ſhe was accordingly visited by Mr. Andrews, surgeon, of Greek-street, along with another gentleman, whose name the patient does not know. On their concurring opinion that her case was incurable, and that nothing could be done for her relief, she at last had recourse to the advice of Mr. Oliphant, of Percy-street; who, with a proper anxiety for his patient, and a zeal at the same time directed by humanity, which every practitioner of medicine should possess, soon after visiting her, and finding that tho' her complaints were palliated, no progress was made towards a cure, advised to call in Dr. Nisbet, whose attention, he understood, had been particularly devoted to this malady.

On my first visit, I found the disease far advanced in its ultimate stage, a short description of which will be sufficient to convince every practitioner of its fatal and apparently speedy termination. An extensive foul spreading ulcer occupied the whole surface of the left breast with thick reverted edges covered with the particular sordes characterizing such sores, and occasionally pouring out quantities of blood from the eroded vessels. Besides the substance of the breast, itself totally diseased, the cuticular glands all round were hard and schirrous to a considerable extent. In the axilla, the glands were swelled to the size of a pigeon's egg, and the whole of that side was tight, contracted, and knotty, with clusters of swelled lymphatics in different parts of it. The patient could hardly use the left arm, and was totally unable to lie on that side. An erysipelatous inflammation diffused itself for a considerable way beyond the actual limits of the disease. On examining the right breast, I found a large schirrous formed in it, which had not yet arrived at an active state. The hectic fever was strongly formed, and the patient's health rapidly declining.

Mr. Oliphant was of opinion the disease was incurable, unless a complete sloughing of the whole breast took place; and I could not, in such a situation, flatter with strong hopes; determined, however, to follow the plan which I have generally found successful, I communicated my sentiments to him, who, with that liberality which every man of real science possesses, acquiesced, and gave every freedom in pursuing it.

The treatment was accordingly begun about the beginning of July, and continued for upwards of six weeks. During its progress, the curative fever was raised to a height sufficient to subdue the morbid action in the diseased parts, and was even attended with some symptoms that required mitigation. Several of the swellings disappeared during the height of the fever; in its progress, the discharge became daily more bland, whitish, and purulent; and immediately on its decline, the sore assumed marks of healing, which have proceeded so rapidly, that it is now within the size of a shilling of being completed. The structure of the breast, (at least what remains of it) is reduced to its natural ſtate; the ſwellings of the arm-pit and ſide are totally removed, and a complete cure in every part ſeems effected.

From the firſt applications that were made, the pain and burning heat from the diſeaſe gradually departed; no ſloughing of any portion took place; but a gradual increaſed abſorption removed the altered organization, and reduced the breaſt to its proper form. This ſhrinking and abſorption were very remarkable long before any marks of healing appeared, and this proceſs ſeemed to be the laſt induced by the action of the remedy.

The above facts will be ſufficient to ſhow, that the principle of cure is different from any that has yet been attempted; but at preſent I ſhall enter into no further detail of it than ſay, that it conſiſts in no ſecret remedy or ſpecific, but proceeds on thoſe general principles which apply to every form and ſtage of the malady.

I have been particular in ſtating the medical advice this patient received, that the Gentlemen mentioned may themſelves make the neceſſary enquiries concerning the iſſue of it.

I am, Gentlemen,

